# Development and Formative Evaluation of a Web-Based Self-Management Exercise and Diet Intervention Program With Tailored Motivation and Action Planning for Cancer Survivors

**DOI:** 10.2196/resprot.2331

**Published:** 2013-02-13

**Authors:** Myung Kyung Lee, Hyeoun-Ae Park, Young Ho Yun, Yoon Jung Chang

**Affiliations:** ^1^Department of NursingDong-A UniversityBusanKorea, Republic Of; ^2^Seoul National UniversityCollege of NursingSeoulKorea, Republic Of; ^3^Seoul National University Hospital and College of MedicineCancer prevention DepartmentSeoulKorea, Republic Of; ^4^National Cancer Control InstituteResearch Institute and HospitalNational Cancer CenterGoyang-siKorea, Republic Of

**Keywords:** Internet, health planning, exercise, diet, self-care, wellness programs

## Abstract

**Background:**

Most dietary and exercise interventions developed to date for cancer survivors have employed intensive clinic-based face-to-face counseling sessions. However, when the clinic-based face-to-face intervention ends, the participants cannot receive feedback from the experts, and the motivation for regular exercise and diet practices decreases. One way to overcome the shortcomings of clinic-based face-to-face intervention is to employ the Internet to this end. To maximize effectiveness when providing Web-based interventions, action planning should be able to start at the right time, education should be tailored to motivational readiness, and self-efficacy should be enhanced at appropriate intervals.

**Objective:**

The aim of this study was to develop a Web-based self-management diet and exercise intervention program with the aid of the transtheoretical model (TTM) and to conduct formative evaluations.

**Methods:**

The Web-based self-management exercise and diet intervention program was developed employing a 5-phase system development life-cycle (SDLC) method. The 5 phases were 1) identification of user requirements, 2) system design, 3) system development, 4) system evaluation, and 5) system application. An expert group composed of 3 content experts, a Web developer, and 2 Web designers, evaluated the usability and accuracy of the content. The program was evaluated by 30 breast cancer survivors for perceived ease of use.

**Results:**

The Web-based self-managed exercise and diet intervention program contained 5 components differing in screen layout. These components are introduction, assessment, education (tailored information provision), action planning (goal setting, scheduling, keeping a diary), and automatic feedback. Education, action planning, and automatic feedback were tailored to each participant through the assessment. The processes of change, self-efficacy, and decisional balance, which are the principal strategies encouraging behavioral change according to the TTM theory, were reflected in the education, and self-efficacy was also reflected in the automatic feedback. After iterative testing by experts on problems that arose in terms of usability and content accuracy during system operation, the perceived ease of use of the program was evaluated by 29 breast cancer survivors. The end users rated the program as being easy to understand and use (a total usability score of 81.3 points). In addition, program feasibility was evaluated using the percentage of patients (27/30, 90%) who consistently used the program.

**Conclusions:**

The use of Internet technology allowed immediate and easy access to interventions, real-time monitoring of progress, online education, tailored action planning, and tailored short message services using mobile phones.

##  Introduction

Survival rates of cancer have improved steadily over the past 30 years [1]. This trend toward improved survival appears likely to continue. Such increases in the number of cancer survivors indicate that quality of life (QOL) issues among survivors must be addressed.

One common health problem in cancer survivors at diagnosis, during treatment, and after treatment is being underweight or overweight. Substantial weight loss has been documented in more than 50% of patients at diagnosis [2]. Symptoms including anorexia, early satiety, changes in taste and smell, and disturbances of the gastrointestinal tract are common side effects of cancer treatment, and such symptoms lead to substantial weight loss [3]. Also, many cancer survivors are overweight or obese at diagnosis. Weight gain (ie, sarcopenic obesity) can be a complication of cancer treatment [4] and is commonly observed during or after treatment of various cancers [3]. In particular, weight gain after cancer diagnosis increases the incidence of subsequent chronic disease, such as cardiovascular disease, diabetes, hypertension, secondary cancers, and cancer recurrence [5]. Both being underweight and overweight negatively affect health related QOL (HRQOL)[6] and survival [7]. For these reasons, maintaining a healthy weight is one of the top priorities when addressing the healthcare needs of cancer survivors [3].

Regular exercise and a balanced diet are essential for healthy weight management. Most dietary and exercise interventions developed to date have employed intensive clinic-based face-to-face counseling sessions [3]. Although the efficacy of such interventions is apparent immediately after delivery, once sufficient time has elapsed, the effects of the intervention cannot be found [8-11] or sustained [12]. The reason is that when the clinic-based face-to-face intervention ends, the participants cannot receive feedback from the experts, and the motivation for regular exercise and diet practices decreases [12]. In addition, the location of the clinic, travel time, and transportation issues are substantial barriers to the successful implementation of clinic-based face-to-face programs [13].

One way to overcome the shortcomings of clinic-based face-to-face intervention is to employ the Internet to this end. To maximize effectiveness when providing Web-based interventions, timely feedback on progress toward desired outcomes should be provided [14], action planning should be able to start at the right time [15], education should be tailored to motivational readiness [16], and self-efficacy should be enhanced at appropriate intervals [17]. The transtheoretical model (TTM) [18] integrates the concepts of self-efficacy and motivation with strategies including tailored feedback and action planning, and arranges these concepts and strategies to guide timely intervention. Therefore, it seemed appropriate to develop a Web-based self-management program using the TTM. The program sought to promote regular exercise and the adoption of a balanced diet to facilitate weight management.

Within the background, the overall aim of this study was to develop a Web-based self-management exercise and diet program featuring the delivery of education, the development of the capacity to plan, automatic feedback employing TTM-based strategies, and to evaluate whether the program was feasible.

##  Methods

### Overview

The Web-based self-management exercise and diet intervention program was developed between February 1 and September 30, 2011, employing a 5-phase system development life-cycle method, which included identification of user requirements, system design, system development, system evaluation, and system application.

###  Phase I: Identification of User Requirements

System requirements included content and functionality. The content requirements of the Web-based self-management exercise and diet intervention program, called Health Planner, was determined via a review of the literature on the diet and exercise requirements of cancer patients after primary treatment. In addition, the development of Health Planner had been referenced to the prior Web-based application, Health Navigation [19], and the this study was part of a larger study—the TTM-based health management program for cancer survivors, called Leadership and Coaching for Health (LEACH). These studies might be applied to empower patients’ ability to take care of themselves in the chronic care model [20]. For LEACH, interviews were conducted from September 2010 to March 2011 using semi-structured questions to seek case information on the healthy habits (ie, positive mindset, regular exercise, healthy diet, regular checkups, no smoking, not overworking), leadership, and commitment possessed by 46 cancer survivors (those who survived more than 5 years after diagnosis). Of the brief notes derived from the interviews, content on exercise and diet habits was extracted and reflected in Health Planner. Before each interview, each participant was informed as to the aim of the project. With semi-structured interviews [21], participants were encouraged to suggest, based on personal experience, information that should be provided to cancer patients. We prioritized information derived from cancer survivors, and selected content items after a consideration of the relevance of such items to our aim of providing effective weight management techniques to cancer survivors.

The functional requirements of the Web-based self-management exercise and diet intervention program were assessed by a review of existing Web-based health management programs for cancer survivors, and were also informed by TTM-based strategies (ie, functions such as stage-matched education, feedback, and action planning).

###  Phase II: System Design

During system design, the content and function of the program were clearly defined with reference to the scope and the objectives. This was achieved by the selection of requirements identified in the first phase. Such clearly defined content and functional requirements were incorporated into the system with the aid of TTM theory. Screen layouts were designed to encompass these; all on-screen were designed with these principles in mind. To render visual communication effectively, and to provide users with a clear and consistent conceptual structure, the user interfaces were designed to be consistent and to be easily navigable.

###  Phase III: System Development

The system was developed on Microsoft Windows (Microsoft, Washington, DC, USA) the Web server application Apache Tomcat 6.0 (Apache Software Foundation, Forest Hill, MD, USA), and the database management system Oracle 10g (Oracle, Redwood Shores, CA, USA) environment. The program was written using Java (Oracle, Redwood Shores, CA, USA) and the JSP Standard Template Library (Sun Microsystems Inc, Santa Clara, CA, USA). Photoshop 8.0 (Adobe Systems, San Jose, CA, USA) and Flash 8 (Adobe Systems, San Jose, CA, USA) were used for Web page design. A short message service (SMS) module was integrated into the program.

###  Phase IV: System Evaluation

In this phase, the usability and accuracy of the content were evaluated by an expert group, composed of 3 content experts (a nutritionist, an exercise physiologist, and a clinical nurse, all of whom had PhDs*),* a Web developer*,* and 2 Web designers. Each component of the program, each element of the content, and all design features were repeatedly tested several times to determine whether any usability problem persisted, whether the content was accurate, if the program had been appropriately modified, and whether the group feedback had been incorporated.

The perceived ease of use of the program was evaluated by 29 breast cancer survivors. Breast cancer patients who had received curative breast cancer surgery with histologically confirmed stage 0-3 cancer were recruited consecutively from participating cancer registries of the 4 study hospitals. These 29 end users completed questionnaires exploring perceptions of the program [22,23]. The scores of items couched in negative terms were reversed; higher scores thus reflect a greater perceived ease of use. Cronbach alpha coefficient was 0.87. In addition, program feasibility was evaluated using the percentage of the patients who consistently used the program for 12 weeks.

### Phase V: System Application

The system developed has been applied to the experimental group of the other interventional study to test program efficacy. The protocol of the intervention study is shown in Multimedia Appendix 1.

##  Results

###  Identifying User Requirements

The content requirements of the Web-based self-managed exercise and diet intervention program were identified by interviewing one key group of cancer patients (who survived more than 5 years after diagnosis) and by reviewing the literature.

Cancer survivors who were questioned about their habits regarding healthy diets reported that they wish to know what to eat and what not to eat. The cancer survivors described the difficulties they faced in maintaining a healthy diet that included at least 5servings of fruits and vegetables (F&V) per day due to lack of preparation time, taste concerns, and fear of pesticide exposure. Several survivors were skeptical about dietary recommendations because information from mass media and research studies often conflicted. However, because most believed that consumption of more F&V afforded overall health benefits, such informational conflicts did not deter the adoption of balanced diets. Some cancer survivors emphasized that it was very difficult to become motivated to change their diet to include more healthy foods. Participants gradually changed their diet over time. Some reported that tight schedules prompted them to eat out more often than they would prefer. They also reported difficulties finding healthy foods in restaurants. A few survivors commented on the high cost of organic vegetables and that F&V spoiled quickly. Even though cost and spoilage were thus identified as barriers, F&V were still consumed.

The main concerns for cancer survivors related to exercise were whether being overweight increases the risk of cancer recurrence, how to exercise during treatment and recovery, and what kinds of special precautions should be taken when exercising. Many women reported that it was difficult to schedule exercise time. A few mentioned that they had followed a routine at one time, but had stopped because cancer treatment had interrupted it or because they lacked an exercise partner. Several barriers rendering inadequate exercise were identified. These included side effects (especially fatigue) of cancer treatment, not having a local gym, and not living near a park. Bad weather sometimes prevented exercise. Some women commented that it was difficult to feel motivated to exercise. Encouragement from friends, or a few words from their doctors, made lasting impressions on them. Participants who discovered a sense of increased energy and a feeling of well-being when exercising were more motivated to maintain physical activity programs. Some female survivors claimed that it was difficult to exercise, particularly when it was dark outside.

Program content requirements obtained by reviewing literature included the need to improve exercise and dietary behavior in cancer survivors [24], the importance of healthy weight management [25,26], barriers to regular exercise and a balanced diet [27], considerations when planning exercise and diet [3], outcomes (such as recurrence, QOL, and survival) associated with the performance of regular exercise and a balanced diet [27,28], exercise and dietary guidelines for cancer survivors [29], and maintenance of regular exercise and balanced diet programs [24].

The functional requirements of the Web-based self-management exercise and dietary intervention program identified by a review of existing Web-based health management programs for cancer survivors were provisions of information, feedback, and evaluation. The functional requirements obtained from a consideration of TTM-based strategies were tailored education, action planning [15,30,31], automatic feedback [14], and comparison between the current status and recommended goal levels of exercise and diet [32].

###  System Design and Development

#### Overall

After clearly defining the content and functional requirements within the scope and objectives of the program, they were arranged and refined with the aid of the TTM theory. The Web-based self-managed exercise and diet intervention program contained 5 components differing in screen layout, which includes the introduction, assessment, education (tailored information provision), action planning (goal setting, scheduling, keeping a diary), and automatic feedback. That is, the program was designed to deliver education, action planning, and automatic feedback relevant to each of the stages of change. Education, action planning, and automatic feedback were tailored to each participant through the assessment. The processes of change, self-efficacy, and decisional balance are the principal strategies encouraging behavioral change according to the TTM theory and were reflected in the education [33-35]. Self-efficacy was also reflected in the automatic feedback. The details of each component of the program are as follows.

#### Introduction

The introduction informed participants of the overall background for developing Health Planner, the usage of the program, and the importance of exercising regularly and eating properly in maintaining good health for cancer survivors.

#### Assessment

The assessment section allowed participants to input their physical activity level, body weight, and stage of motivational readiness. All participants were screened for any contraindications to exercise using the physical activity readiness questionnaire [36] during assessment. Using algorithms based on input data, each patient could access tailored information appropriate to each stage of change, and was prescribed the appropriate number of portions of 6 food groups given their physical activity level and body mass index (BMI, measured on a daily basis).

#### Education

Each participant was scheduled to be online for 5-10 minutes each week. The educational content was divided into 5 modules based on the current stage of motivational readiness of each patient through assessment [18]. For patients in the precontemplation stage, education focused on raising consciousness, dramatic relief, environmental reevaluation, and increasing the number of pros. For patients in the contemplation stage, education focused on self-reevaluation, increasing the number of pros, decreasing the number of cons, and building self-efficacy. For patients in the preparation stage, education focused on self-liberation, and remembering and increasing the number of pros. For patients in the action and maintenance stages, education focused on reinforcement, assisting with relationships, counter-conditioning, stimulus control, and management of temptation.

#### Action Planning

##### Components

The action planning included setting a recommended goal, planning, keeping a diary, and comparing between current and recommended levels of exercise and diet. Each participant was encouraged to actively plan their exercise behavior in line with the American Cancer Society (ACS) guidelines for cancer survivors [24], and to achieve an excellent dietary score (measured using the Korean version of the Diet Quality Index). The details of exercise and diet planning delivered via Health Planner are as follows.

##### Exercise Planning

The goal of exercising was to perform at least moderate-intensity aerobic exercise for at least 30 minutes on at least 5 days each week (to yield 12.5 metabolic equivalents of energy expenditure, in line with ACS guidelines for cancer survivors) [24]. Health Planner generated a tailored plan for each participant through assessment. If a patient had no history of exercise prior to cancer treatment, exercise was gradually introduced [3]. Planning regular exercise was set to start at the preparation stage. The exercise was to be aerobic in nature, and the specific type of exercise was based on individual patient preference. The type, intensity, duration, and frequency of exercise could be self-adjusted as necessary depending on patient age, history of exercise, and subjective experience of tiredness. Exercise plan was implemented as an event on a calendar.

##### Dietary Planning

The goal of dietary planning was to achieve an excellent dietary quality score (measured using the Diet Quality Index [37]). The aims included an energy level derived from fat of ≤20%, an energy level derived from saturated fat of ≤6%, cholesterol ≤300 mg/day, an energy level derived from carbohydrates of ≤65%, an intake of vegetables and fruit of ≥7 servings/day, a protein recommended dietary allowance of 75-125%; a calcium recommended dietary allowance of 75-125%; and a sodium intake of ≤3500 mg/day. Dietary planning was based on individual BMI values, ideal body weights, and daily calorific requirements. Each patient was educated in terms of the recommended daily number of portions from the 6 food groups (grain, meat/fish/eggs/beans, vegetables, fruit, milk and dairy products, and fats and oils) as suggested by the Korean Nutrition Society (2010). All participants were encouraged to achieve a balanced diet.

Participants recorded the daily number of portions of 6 food groups consumed in a dietary diary and daily exercise behavior (type, intensity, and duration) in an exercise diary. These data were used to give automatic feedback on progress toward goal attainment (the SMS module was employed toward this end). The data were also presented visually where a graph compared the actual amount of exercise done, dietary intake, and the behaviors to what were recommended.

#### Automatic Feedback

Participants were asked to input, on a daily basis, the number of portions from the 6food groups consumed and the details of their exercise behavior (type, intensity, and duration of exercise) as shown in the exercise and dietary diaries. This information was used to provide feedback on progress toward goal attainment in the SMS module. Comparisons of the daily number of portions from the 6 food groups consumed with the recommended number, and of the weekly energy expenditure on aerobic exercise with the exercise goal identified patients who attained goal behaviors. These patients were given immediate reinforcement via positive automated messaging. Patients who did not attain goal behavior were encouraged to restart active exercise or to increase their dietary efforts. Such patients were encouraged to increase their level of physical activity or to attain a balanced diet by increasing F&V intake or moving to a low-carbohydrate or low-fat diet.

The protocol on the interventional goal, principal strategies, content theme in the educational component, and functions used for delivering interventions at each stage of change are briefly summarized in Table 1.

####  System Evaluation

In this phase, experts who had participated in the system design and development were contacted again and asked to advise on problems that arose in terms of usability and content accuracy during system operation. For example, the confusing array of content was rearranged to ensure consistency and relevance. Input speed was improved. The functions (ie, keeping a diary, setting a weekly exercise goal, measuring weekly body weight for a revised diet prescription, measuring the stage of change, SMS-based feedback) that depend on the stage of change and timing were modified to activate at an appropriate stage or timing. Images of various food servings were added and an example of the written 3-day dietary recall report was included. The number of pop-ups (negatively affecting concentration) was reduced. Tasks that were shown as incomplete on the calendar were identified. An SMS alarm was added to inform patients of the weekday on which education would be given. In addition, various bugs and errors were corrected. After iterative testing, the program was modified and installed on a server. Patients could access Health Planner from a home computer using an Internet browser.

The characteristics of end users participating in the usability evaluation are shown in Table 2. The end users rated the program as being easy to understand and use (a total usability score of 81.3 points, Table 3). In addition, program feasibility was evaluated using the percentage of patients (27/30, 90%) who consistently used the program for 12 weeks.

**Table 1:** Protocol of the Web-based self-management exercise and diet intervention program.

Stage of change	Interventional goal	Principal strategies (process of change, self-efficacy, decisional balance)	Content theme in the educational component	Functions used for delivering interventions
Precontemplation	Increase awareness of the need to change exercise and dietary behavior	Consciousness-raising Dramatic relief Environmental reevaluation Increase the pros	Effect of exercise and a balanced diet on health Specific reasons for not considering exercise or use of a balanced diet Risks associated with a sedentary lifestyle and an unbalanced diet	Weekly Web-based tailored education (5-10 minutes daily).
Contemplation	Motivate and increase confidence in the ability to change; build motivation for change	Self reevaluation Increase the pros/decrease the cons Build self-efficacy	Specific benefits of exercise and use of a balanced diet; barriers toward such achievements Solutions to overcome the specific barriers to exercise and use of a balanced diet Contemplation of improved health following exercise and use of a balanced diet	Weekly Web-based tailored education (5-10 minutes daily)
Preparation	Develop and negotiate a plan for exercise and use of a balanced diet	Self-liberation Remember the pros Increase self-efficacy	Recalling the effects of exercise and a balanced diet on health Individualized exercise and dietary prescription and creation of specific aims of exercise Keeping and monitoring of a daily exercise and dietary diary Planning gradual progress in terms of exercise Exercise planning in line with ACS	Weekly Web-based tailored education (5-10 minutes daily) Use of an exercise and diet diary Weekly planning exercise and diet
Action	Reaffirm the commitment to exercise and to use of a balanced diet	Reinforcement management Assisting with relationships Counter-conditioning Stimulus control Managing of temptation	Evaluation of current exercise and dietary pattern Self-reward for regular exercise and use of a balanced diet Recall of the specific aims or reasons for performing regular exercise and using a balanced diet Substitution of exercise for sedentary behavior and a balanced diet for one that was unbalanced Social and/or family support to help maintain exercise and dietary programs Avoidance of stimuli and other triggers provoking inactivity or use of an unbalanced diet Individualized exercise and dietary prescription detailing the specific aims of exercise Keeping and monitoring of a daily exercise and dietary log Planning gradual progress toward more exercise Exercise planning in line with ACS	Weekly Web-based tailored education (5-10 minutes daily) Use of an exercise and diet diary Weekly planning of exercise and diet Daily or weekly feedback on progress toward goal attainment Comparison of the current status and the goal levels of exercise and diet
Maintenance	Develop strategies to prevent relapse			

**Table 2:** Characteristics of end users participating in the usability evaluation.

Characteristic		Participants N=30 n (%)
**Age in year**		
	Mean (SD)	41.5 (6.3)
**Educational level**		
	High school	7 (23)
	College or beyond	23 (77)
**Marital status**		
	Married	27 (90)
	Not married	3 (10)
**Time elapsed since treatment, days**		
	Mean (SD)	161.6 (107.8)
	Range	26–349
**Surgery type**		
	Breast-conserving	20 (67)
	Mastectomy	10 (33)
**Receiving chemotherapy**		
	No	4 (13)
	Yes	26 (87)
**Receiving radiotherapy**		
	No	3 (10)
	Yes	27 (90)
**Clinical stage**		
	Stage 0	2 (7)
	Stage I	12 (40)
	Stage II	13 (43)
	Stage III	3 (10)

**Table 3:** Usability evaluation of health planner by end user responses.

		N=30 Mean (SD)^a^
**Items**		
	1. I thought this program was easy to understand.	5.9 (1.4)
	2. I could complete the tasks that were asked of me in this program.	5.7 (1.4)
	3. I found this program confusing.	1.5 (1.1)
	4. I thought that this program was easy to use.	5.9 (2.0)
	5. I would choose to use this type of program in the future to complete an intervention that aims to improve my health.	6.5 (1.5)
	6. The program was too complex.	2.2 (1.3)
	7. I would need help from a technical support person to be able to use this program.	1.3 (1.8)
	8. The program ran smoothly.	6.0 (1.7)
	9. The program was inconsistent (there were parts of the program that seemed out of place).	2.0 (1.3)
	10. I think that most people would learn to use this program quickly.	5.7 (1.3)
	11. Using this program felt awkward to me.	1.9 (1.4)
	12. I felt very confident using this program.	6.0 (1.8)
	13. I needed to learn a lot of things before I could get going with this program.	2.4 (1.6)
**Total Usability Score** ^b^		81.3 (20.2)

^a^Responses were on a 7-point scale, ranging from 1 (strongly disagree) to 7 (strongly agree).

^b^This is a composite of the responses to all usability questions (a 100-point score); higher scores indicate greater perceived usability.

##  Discussion

There is a widely recognized need for interventions to increase healthy behaviors for cancer survivors. However, the effectiveness of traditional interventions remains unclear due to low accessibility and non-persistent feedback. We developed the Web-based self-management exercise and dietary intervention program aiming to increase exercise and improve dietary behavior among cancer survivors. The program has several notable features. Critically, we embraced action planning when considering exercise and dietary behavior. The Web-based self-management exercise and diet intervention program managed day-to-day health behavior via action planning. Practical and specific action planning and scheduling promotes the initiation and maintenance of healthy behavior by identifying and filling intention-behavior gaps [15].

The program provided timely reinforcement via daily delivery of positive automated messages. The tailored feedback-based intervention may provide relief to survivors in that someone (or something) close to the patients is managing their health [38], motivating the patients to achieve and maintain goal behaviors at the recommended levels. Earlier work suggested that maintenance of healthy behavior is greatly assisted by the provision of appropriate feedback on progress toward desired outcomes [14]. The tailored reaction might stimulate self-regulatory behavior through making self-judgments on progress toward desired exercise and diet behavior [39].

The patients’ needs were reflected in the program through interviews with the patients, and the identified user requirements were combined with technologies to implement TTM theory-based functions. Our Web-based self-management program, featuring the use of TTM theory may not only trigger the required behaviors, but may assist in sustaining such long-term behaviors [12,16]. Self-regulation strategies, including diary keeping, an emphasis on goal-setting, and feedback on progress may increase motivation and perceived self-efficacy [33].

A review of previous studies identified the possibility that the TTM could be applied to a Web-based program seeking to encourage healthy behavioral changes. The potential of TTM theory has been recognized in previous studies for application in Web-based physical activity intervention [40], but not for Web-based diet programs. Individual exercise regimens were prescribed using TTM theory, with reference to stages of change, in an interventional Web-based program developed for patients with type 2 diabetes [40]. Program clarity, simplicity, recommendations, accuracy, consistency, and efficiency were rated as acceptable. In another study, a Web-based TTM-based program was developed to promote physical activity in the general female population [41]. The program was a full success, having promoted self-efficacy, physical activity, and exercise. To date, however, no Web-based program aimed at such lifestyle modifications in cancer survivors is yet available.

Previous findings of Web-based lifestyle intervention trials remain controversial. Delivery of theory-based messages via the Web enhanced lifestyle modifications [19,40,42]. However, a Web-based program that apparently failed to trigger lifestyle changes lacked a theoretical basis, had low utilization, and did not suit participants’ needs [43]. This program rated high utilization. It might be due to the strategies of daily feedback and action planning. The diagnosis of cancer also provides a teachable moment when the patients’ motivation for lifestyle change is especially high [44].

In conclusion, a Web-based program targeting change in exercise and dietary behaviors was feasible when TTM theory was used to inform program strategy. The use of Internet technology allowed immediate and easy access to interventions, real-time monitoring of progress, online education, tailored action planning, and tailored SMS using mobile phones [45]. Most healthcare providers in busy clinical settings rarely find time to counsel patients on health management. Given that the number of cancer survivors is increasing, targeting of such high-risk groups can potentially achieve positive and widespread public health outcomes.

## Multimedia Appendix 1

The protocol of the trial.

## Abbreviations

ACS: American Cancer Society
BMI: body mass index
F&V: fruits and vegetables
HRQOL: health related quality of life
LEACH: Leadership and Coaching for Health
QOL: quality of life
SMS: short message service
TTM: transtheoretical model
