# Supplementary material for: Development and Formative Evaluation of a Web-Based Self-Management Exercise and Diet Intervention Program With Tailored Motivation and Action Planning for Cancer Survivors
Source: JMIR Res Protoc. 2013 Feb 13;2(1):e11. doi: 10.2196/resprot.2331 (PMC3628152; doi:10.2196/resprot.2331)
Supplement: Supplementary file 1 [file resprot_v2i1e11_app1.pdf]

Multimedia Appendix 1

Study Protocol

Efficacy of a Web-based Tailored Self-management Program

Purpose

Lifestyle interventions that promote exercise and a healthy diet, which have the potential to improve health-related quality of life (HRQOL), may be particularly appropriate for cancer survivors. The previous studies suggested that a key strategy to implement sustainable healthy behavior and improve health was providing appropriate feedback and promoting self-efficacy. Web-based program enables to provide the feedback in a timely manner on a daily basis, to continue the healthy behaviors.

Therefore, the investigators developed a web-based, stage-matched Exercise and Diet Planning program, and whether the program can promote significantly greater changes in behavioral outcomes [goal of exercise (energy expenditure of aerobic exercise ≥12.5 kcal/kg/week) and diet (intake of vegetables ≥5 serv/day and intake of fruit = 1-2 serv/day)], stage of changes for exercise and diet, psychosocial outcomes (HRQOL, fatigue, anxiety and depression) and self-efficacy in implementing goal of exercise and diet among breast cancer survivors in Korea was examined.

Hypotheses were following:

1. Survivors of a group participating in a web-based, stage-matched Exercise and Diet Planning program (hereinafter called the 'intervention group') will show a more advanced stage of change for exercise and diet compared to survivors in the control group.

2. The intervention group will show a higher proportion of attaining goal of exercise (or higher level of energy expenditure of aerobic exercise) compared to the control group.

3. The intervention group will show a higher proportion of attaining goal of diet (or higher level of diet quality) compared to the control group.

4. The intervention group will show a better HRQOL level compared to the control group.

5. The intervention group will show a better self-efficacy level compared to the control group.

6. The intervention group will show a lower fatigue level compared to the control group.

7. The intervention group will show less anxiety compared to the control group.

8. The intervention group will show less depression compared to the control group.

Conditions: Breast Cancer

Intervention: Behavioral: Web-based Health Planner on diet and exercise

Control: Behavioral: Non-tailored booklet on exercise and diet

Phase: N/A

Study Type: Interventional

Study Design: Supportive Care, Parallel Assignment, Single Blind (Outcomes Assessor), Randomized, Efficacy Study

Official Title: Efficacy of a Web-based Self-management Exercise and Diet Intervention Program With Tailored Motivation and Action Planning for Breast Cancer Survivor: A Randomized Controlled Trial

Primary Outcome Measure:

Change from baseline in numbers of goal behaviors at 3 months [Time Frame: Baseline (pre-treatment) and after 12 weeks of intervention (post-treatment)]

The primary outcomes of the study are to achieve goals of healthy behaviors as followed:

i) exercising≥12.5kcal/kg/week;

ii) eating vegetables≥5serv/day and fruits 1-2 serv/day;

iii) healthy weight (18.5kg/m2 ≤BMI < 25kg/m2)

The primary outcome of the study is the increased number of goal behaviors.

Secondary Outcome Measures:

Change from baseline in Stage of Change at 3 months [Time Frame: Baseline, 1month, 2 month, 3 month after starting the intervention]: Stage of motivational readiness for exercise and diet based on the established TTM

Change from baseline in self-efficacy at 3 months [Time Frame: Baseline, 1month, 2 month, 3 month after starting the intervention]: The self-efficacy for exercising≥12.5kcal/kg/week, eating vegetables≥5serv/day and fruits 1-2 serv/day

Change from baseline in psychosocial outcomes at 3 months [Time Frame: Baseline, 1month, 2 month, 3 month after starting the intervention]: The psychosocial outcomes are HRQOL, fatigue, anxiety and depression

Change from baseline in Diet quality at 3 months [Time Frame: Baseline, 1month, 2 month, 3 month after starting the intervention]: Diet quality based on a three-day diet recall and the Diet Quality Index (DQI) revised for the Korean population

Enrollment: 59

Study Start Date: October, 1st, 2011

Arms

Experimental: Web-based, Stage-matched Exercise and Diet Planning program

The experimental arm is a group that assigned to use web-based, stage-matched exercise and diet planning program.

Active Comparator: Non-tailored booklet on exercise and diet

The control group is provided a booklet containing same information on exercise and diet as in the experimental group's web-based program, but the information

on a booklet is not tailored to participants' stage of motivational readiness for exercise and diet based on the TTM.

Assigned Interventions

Behavioral: Web-based Health Planner on diet and exercise

URL: http://healthplanning.ncc.re.kr

The program is 12-week program, provides tailored information on the exercise and diet based on the stage of motivational readiness of TTM. The program is designed to allow to plan a regular exercise of 12.5 MET per week and to recommend to eat number of portions from six food groups for balanced diet tailored to individual's BMI, ideal body weight, and calories needed per day. The program provides the feedback based on the achievement of goals of the behaviors.

Other Names:

English name: Health Planner

Korean name:

Behavioral: Non-tailored booklet on exercise and diet

The active control group is provided non-tailored booklet on exercise and diet, whose information is same as web-based program except stage-matching.

The purpose of the study was to develop a web-based, stage-matched Exercise and Diet Planning program and to examine effects of the program on implementation of exercise and diet, self-efficacy, HRQOL, fatigue, anxiety and depression among breast cancer survivors.

All participants were recruited from two tertiary university hospitals and the National Cancer Center in Korea.

Eligibility

Ages Eligible for Study: 20 years and older

Genders Eligible for Study: Female

Inclusion Criteria:

1. Women aged 20 years and older

2. Histologically confirmed stage 0 to III breast cancer who received curative breast cancer surgery within 12 months since completion of primary cancer treatment and 2 years since diagnosis

3. Serum platelet $\geq$100,000/mm^3

4. Serum hemoglobin $\geq$10g/dl

5. Not met one or more behavior goals: i) energy expenditure of aerobic exercise $\geq$ 12.5 kcal/kg/week; ii) intake of vegetables except kimchi $\geq$5 serv/day and intake of fruit 1-2 serv/day; iii) maintenance of healthy weight (18.5 kg/m^2 $\leq$BMI < 23 kg/m^2)

6. Ability to use internet, and being accessible internet at home

7. Mobile phone user

8. Consent form to participate in the study

Exclusion Criteria:

1. Currently receiving any cancer treatment

2. Conditions that could limit adherence to an unsupervised exercise program such as uncontrolled congestive heart failure or angina, recent myocardial infarction, or breathing difficulties requiring oxygen use or hospitalization; walker or wheelchair use; or plans to have hip or knee replacement

3. Conditions that could interfere with a high vegetable and fruit diet, such as kidney failure or chronic warfarin use

4. Progressive malignant disease or additional primary cancers

5. Infectious condition (body temperature $\geq$ 37.2 or WBC $\geq$ 11,000 mm^3)

6. Inability to use computer or internet

7. Visual and motor dysfunction
